# Screening trials of spinal cord stimulation for neuropathic pain in England—A budget impact analysis

**DOI:** 10.3389/fpain.2022.974904

**Published:** 2022-09-06

**Authors:** Rui V. Duarte, Rachel Houten, Sarah Nevitt, Morag Brookes, Jill Bell, Jenny Earle, Ashish Gulve, Simon Thomson, Ganesan Baranidharan, Richard B. North, Rod S. Taylor, Sam Eldabe

**Affiliations:** ^1^Liverpool Reviews and Implementation Group, University of Liverpool, Liverpool, United Kingdom; ^2^Saluda Medical Pty Ltd., Artarmon, NSW, Australia; ^3^The James Cook University Hospital, Middlesbrough, United Kingdom; ^4^Patient and Public Involvement Representatives, Middlesbrough, United Kingdom; ^5^Department of Pain Medicine and Neuromodulation, Mid and South Essex University Hospitals, Essex, United Kingdom; ^6^Leeds Neuromodulation Centre, Leeds Teaching Hospitals, Leeds, United Kingdom; ^7^Neurosurgery, Anesthesiology and Critical Care Medicine (ret.), Johns Hopkins University School of Medicine, Baltimore, MD, United States; ^8^College of Medicine and Health, University of Exeter, Exeter, United Kingdom; ^9^MRC/CSO Social and Public Health Sciences Unit and Robertson Centre for Biostatistics, Institute of Health and Well Being, University of Glasgow, Glasgow, United Kingdom

**Keywords:** budget impact analysis, spinal cord stimulation, screening trials, neuropathic pain, cost savings

## Abstract

Screening trials of spinal cord stimulation (SCS) prior to full implantation of a device are recommended by expert guidelines and international regulators. The current study sought to estimate the budget impact of a screening trial of SCS and the costs or savings of discontinuing the use of a screening trial. A budget impact analysis was performed considering a study population that reflects the size and characteristics of a patient population with neuropathic pain in England eligible for SCS. The perspective adopted was that of the NHS with a 5-year time horizon. The base case analysis indicate that a no screening trial strategy would result in cost-savings to the NHS England of £400,000–£500,000 per year. Sensitivity analyses were conducted to evaluate different scenarios. If ≥5% of the eligible neuropathic pain population received a SCS device, cost-savings would be >£2.5 million/year. In contrast, at the lowest assumed cost of a screening trial (£1,950/patient), a screening trial prior to SCS implantation would be cost-saving. The proportion of patients having an unsuccessful screening trial would have to be ≥14.4% for current practice of a screening trial to be cost-saving. The findings from this budget impact analysis support the results of a recent UK multicenter randomized controlled trial (TRIAL-STIM) of a policy for the discontinuation of compulsory SCS screening trials, namely that such a policy would result in considerable cost-savings to healthcare systems.

## Introduction

Screening trials before implantation of a spinal cord stimulation (SCS) device began in the early 1970's (1, 2), shortly after the first reported use of SCS for pain treatment (3). Screening trials to test patients' responses and if the stimulation is well-tolerated consist of insertion of a temporary or permanent lead attached to an external stimulator. Current international guidelines variously define a successful screening trial as ≥50% pain relief and satisfactory on table paraesthesia coverage (i.e., ≥80%) of the pain area for paraesthesia inducing stimulation and/or reduction in pain medications or improved health-related quality of life and function, and/or successful location of leads at anatomical target for paraesthesia free therapies (4, 5). Randomized controlled trials (RCTs) that evaluated the efficacy of SCS for pain-related conditions have all required that patients undergo a screening trial to evaluate early response to therapy prior to full implantation of the SCS device (6–12). For some indications such as chronic anginal pain (13), or for patients with higher risk of infection or bleeding, a trial may not need to be performed.

Although a successful screening trial has been widely accepted by the majority of the clinical community as a predictor of long-term response to SCS, the TRIAL-STIM RCT showed that a screening trial does not provide superior patient outcomes and is not cost-effective compared to not doing a screening trial (14). Long-term results at 36-month follow-up were consistent with the RCT primary endpoint findings (15). Qualitative results of TRIAL-STIM also indicated that patients overwhelmingly preferred not to undergo a trial (16). Downsides to screening trials include higher infection rates particularly for trials with longer durations (17, 18).

The economic burden of screening trials for healthcare budgets has been less explored. A previous cost-analysis estimated that cost-savings could be made by adopting a SCS implantation strategy without a screening trial (19). However, the authors of this analysis highlighted its limitations (a modeling exercise study based on the extrapolation of screening trial to successful implant rates reported in RCTs) and that further research was required to confirm their findings.

The aim of this study is to conduct a budget impact analysis to estimate the costs or savings of discontinuing the compulsory use of a screening trial before implantation of a SCS device.

## Methods

The methods follow the principles outlined in the National Institute for Health and Care Excellence (NICE) guidelines to assess budget impact (20). The budget impact analysis was conducted from the perspective of the NHS with a 5-year time horizon.

### Study population

The study population reflects the size and characteristics of a patient population with neuropathic pain in England currently considered for SCS. A previous study retrieved Hospital Episode Statistics (HES) data to estimate the number of patients with neuropathic pain potentially eligible for SCS and patients that received an SCS related procedure in the NHS in England up to 2020 (21).

Current NICE and international regulatory guidance recommend a screening trial prior to full implant (4, 5, 22, 23). Therefore we considered for the base case analysis that 100% of patients would have a screening trial before implantation of the SCS device. A sensitivity analysis was conducted to account for HES data estimates that not all patients implanted with a SCS device have a screening trial in NHS practice (21). A failure rate of 10.6% for a screening trial was taken from the TRIAL-STIM RCT, conducted in three large implanting centers, that reflected routine clinical practice in England (14).

### Time horizon

To estimate the future budget impact of current practice which includes a SCS screening trial in comparison to an alternative strategy where a screening trial would be discontinued, the neuropathic pain population growth from 2010/11 to 2018/19 was used to estimate the neuropathic pain population potentially eligible for an SCS in the subsequent 5 years. The average proportion of the total eligible population undergoing SCS procedures from 2014/15 to 2018/19 was used for each subsequent year as it remained constant over this period at 0.9% (21). We did not use the NHS financial year end of 2019/20 as it is likely that several hospitals had reduced elective pain activity up to April 2020 as a part of pain staff repurposing efforts due to the COVID-19 pandemic, reducing national rates of neuropathic pain related procedures and SCS implants.

### Costs

The intervention costs used are outlined in Table 1. Costs considered in the analysis included screening trial, implantation of the implantable pulse generator (IPG) and costs associated with electrode removal due to an unsuccessful screening trial. Device costs were considered for a rechargeable IPG. Costs were not discounted over time for the purposes of the budget impact analysis (20). Where required, prices were inflated to the 2020 price year using Personal Social Services Research Unit (PSSRU) Pay and Prices Index (24).

**Table 1:** Procedure and device costs.

**Procedure or device**	**Base-case value**	**Range**	**Source**
SCS related procedures			
SCS trial	£2,687	£1,920 to £4,975	NHS (2019-20) AB14Z, (24) NICE (25)
Insertion of Neurostimulator for Pain Management	£3,877	-	NHS (2019-20) AB12Z (24)
Unsuccessful SCS trial (Electrode removal)—perm leads	£2,628	-	Simpson et al. (26)*
Temporary lead removal	-	£192 to £458	NHS (2019-20) WF01A, HC65Z (24)
SCS IPG			
Rechargeable SCS IPG	£17,422	£13,726 to £22,418	NICE (25)

IPG, implantable pulse generator; SCS, spinal cord stimulation.

*Inflated to 2020 price year.

### Data analysis

We conducted a base case analysis and sensitivity analyses. The base case analysis assumed that every new patient would have a screening trial prior to implantation of the SCS device and the base case costs for procedures and device as presented in Table 1. Sensitivity analyses were conducted to reflect:

1. The rate of screening trials as observed in the HES data were used as opposed to the 100% screening trial rate used in the base case to reflect current recommendations (4, 5, 22, 23).
2. The proportion of the eligible neuropathic pain population that receive an SCS was increased to 5%.
3. The screening trial cost was varied to the upper and lower bounds (25).
4. The rechargeable SCS device cost was varied to the upper and lower bounds (25).
5. The rate of screening trial failure was varied to estimate at what point current practice would become cost-saving.
6. Screening trials conducted with temporary leads for all patients.
7. Screening trials conducted with temporary leads for 30% of the patients (14).
8. Screening trials conducted with temporary leads for 30% of the patients reflecting the rate of screening trials as observed in the HES data.

The base case and the sensitivity analyses examined the budget impact as the difference in total costs between current practice and a potential future scenario where a screening trial is not required prior to SCS device implantation.

## Results

The results considering the figures for the year 2019/20 are presented in Table 2. A no screening trial strategy results in higher SCS device costs and implant costs, however this is offset by the screening trial costs and unsuccessful screening trial costs incurred in current practice. A no screening trial cost strategy would result in a cost-saving of £403,394 for the year 2019/20.

**Table 2:** Difference in current practice and a no screening trial strategy for the year 2019/20.

	**Current practice**	**No screening trial**
No. patients with SCS	576	576
No. patients with a screening trial	576	-
No. patients with unsuccessful screening trial	61	-
SCS device costs	£8,967,511	£10,035,072
SCS implant costs	£1,995,620	£2,233,194
Screening trial costs	£1,547,520	-
Unsuccessful screening trial costs	£161,009	-
Total	£12,671,660	£12,268,266
Cost difference	£403,394	
Cost difference per patient	£700	

SCS, spinal cord stimulation.

Considering the projected increase in prevalence of neuropathic pain in England (1.035) and related increase in the number of new SCS implants for the subsequent years, the cost-savings associated with a no screening trial strategy are expected to increase, reaching close to £500,000 in cost-savings per year from 2023/24 onwards (Table 3).

**Table 3:** Difference in current practice and a no screening trial strategy from 2020/21 to 2024/25.

**Year**	**2020/21**	**2021/22**	**2022/23**	**2023/24**	**2024/25**
No. patients with SCS	619	640	663	687	711
No. patients with a screening trial	619	640	663	687	711
No. patients with unsuccessful screening trial	66	68	71	73	76
Cost difference between current practice and no screening trial (per patient ~£700)	£433,189	£448,534	£464,421	£480,872	£497,905

SCS, spinal cord stimulation.

### Sensitivity analysis

To test the robustness of the results to the uncertainty in the parameters within the budget impact model, a number of sensitivity analyses were conducted. The scenario analysis indicates that scenarios using the current practice in England as suggested by the HES data where not all patients received a screening trial, an increase in the proportion of the eligible neuropathic pain population that receive an SCS and alternative SCS device costs continues to result in cost-savings for a no screening trial strategy (Table 4). The cost-savings achieved could be over £2.5 million per year if at least 5% of the eligible neuropathic pain population received a SCS device. Only a scenario where the cost of a screening trial is at the lower value of £1,920 would result in current practice of a screening trial prior to SCS implantation being cost-saving. The upper value of a screening trial of £4,975 would result in cost-savings ranging from £1.8 million in 2020/21 to £2.1 million in 2024/25. Current practice of a screening trial prior to SCS implantation would be cost-saving if 14.4% or more of the patients considered for SCS had an unsuccessful screening trial.

**Table 4:** Sensitivity analysis results.

**Year**	**2020/21**	**2021/22**	**2022/23**	**2023/24**	**2024/25**
Rate of screening trials as observed in the HES data					
No. patients with SCS	619	640	663	687	711
No. patients with a screening trial	500	518	536	555	575
No. patients with unsuccessful screening trial	53	55	57	59	61
Cost difference between current practice and no screening trial (per patient ~£566)	£350,244	£362,651	£375,496	£388,797	£402,569
Proportion of the eligible neuropathic pain population that receive an SCS increased to 5%					
No. patients with SCS	3,436	3,558	3,684	3,815	3,950
No. patients with a screening trial	3,436	3,558	3,684	3,815	3,950
No. patients with unsuccessful screening trial	366	379	392	406	420
Cost difference between current practice and no screening trial (per patient ~£700)	£2,406,607	£2,491,853	£2,580,118	£2,671,510	£2,766,139
Screening trial cost varied to the lower bound value					
No. patients with SCS	619	640	663	687	711
No. patients with a screening trial	619	640	663	687	711
No. patients with unsuccessful screening trial	66	68	71	73	76
Cost difference between current practice and no screening trial (per patient ~£66)	-£41,028	-£42,481	-£43,986	-£45,544	-£47,157
Screening trial cost varied to the upper bound value					
No. patients with SCS	619	640	663	687	711
No. patients with a screening trial	619	640	663	687	711
No. patients with unsuccessful screening trial	66	68	71	73	76
Cost difference between current practice and no screening trial (per patient ~£2,986)	£1,848,625	£1,914,106	£1,981,907	£2,052,109	£2,124,798
Rechargeable SCS device cost varied to the lower bound value					
No. patients with SCS	619	640	663	687	711
No. patients with a screening trial	619	640	663	687	711
No. patients with unsuccessful screening trial	66	68	71	73	76
Cost difference between current practice and no screening trial (per patient ~£1,093)	£676,396	£700,355	£725,162	£750,849	£777,445
Rechargeable SCS device cost varied to the upper bound value					
No. patients with SCS	619	640	663	687	711
No. patients with a screening trial	619	640	663	687	711
No. patients with unsuccessful screening trial	66	68	71	73	76
Cost difference between current practice and no screening trial (per patient ~£169)	£104,440	£108,139	£111,969	£115,936	£120,042
Rate of screening trial failure varied to the point current practice would become cost-saving (14.4%)					
No. patients with SCS	619	640	663	687	711
No. patients with a screening trial	619	640	663	687	711
No. patients with unsuccessful screening trial	89	92	95	99	102
Cost difference between current practice and no screening trial (per patient ~£2)	-£1,255	-£1,300	-£1,346	-£1,393	−1,443
Screening trials conducted with temporary leads for all patients (lower bound value)					
No. patients with SCS	619	640	663	687	711
No. patients with a screening trial	619	640	663	687	711
No. patients with unsuccessful screening trial	66	68	71	73	76
Cost difference between current practice and no screening trial (per patient ~£613)	£379,324	£392,760	£406,672	£421,077	£435,992
Screening trials conducted with temporary leads for all patients (upper bound value)					
No. patients with SCS	619	640	663	687	711
No. patients with a screening trial	619	640	663	687	711
No. patients with unsuccessful screening trial	66	68	71	73	76
Cost difference between current practice and no screening trial (per patient ~£878)	£543,311	£562,556	£582,483	£603,115	£624,478
Screening trials conducted with temporary leads for 30% of the patients (lower bound value)					
No. patients with SCS	619	640	663	687	711
No. patients with a screening trial	619	640	663	687	711
No. patients with unsuccessful screening trial	66	68	71	73	76
Cost difference between current practice and no screening trial (per patient ~£674)	£417,030	£306,483	£447,097	£462,933	£479,331
Screening trials conducted with temporary leads for 30% of the patients (upper bound value)					
No. patients with SCS	619	640	663	687	711
No. patients with a screening trial	619	640	663	687	711
No. patients with unsuccessful screening trial	66	68	71	73	76
Cost difference between current practice and no screening trial (per patient ~£753)	£466,226	£482,740	£499,840	£517,545	£535,877
Screening trials conducted with temporary leads for 30% of the patients and rate of screening trials as observed in the HES data (lower bound value)					
No. patients with SCS	619	640	663	687	711
No. patients with a screening trial	500	518	536	555	575
No. patients with unsuccessful screening trial	53	55	57	59	61
Cost difference between current practice and no screening trial (per patient ~£545)	£337,179	£349,122	£361,489	£374,293	£387,551
Screening trials conducted with temporary leads for 30% of the patients and rate of screening trials as observed in the HES data (upper bound value)					
No. patients with SCS	619	640	663	687	711
No. patients with a screening trial	500	518	536	555	575
No. patients with unsuccessful screening trial	53	55	57	59	61
Cost difference between current practice and no screening trial (per patient ~£609)	£376,955	£390,308	£404,133	£418,448	£433,270

HES, hospital episode statistics; SCS, spinal cord stimulation.

**Figure 1:**
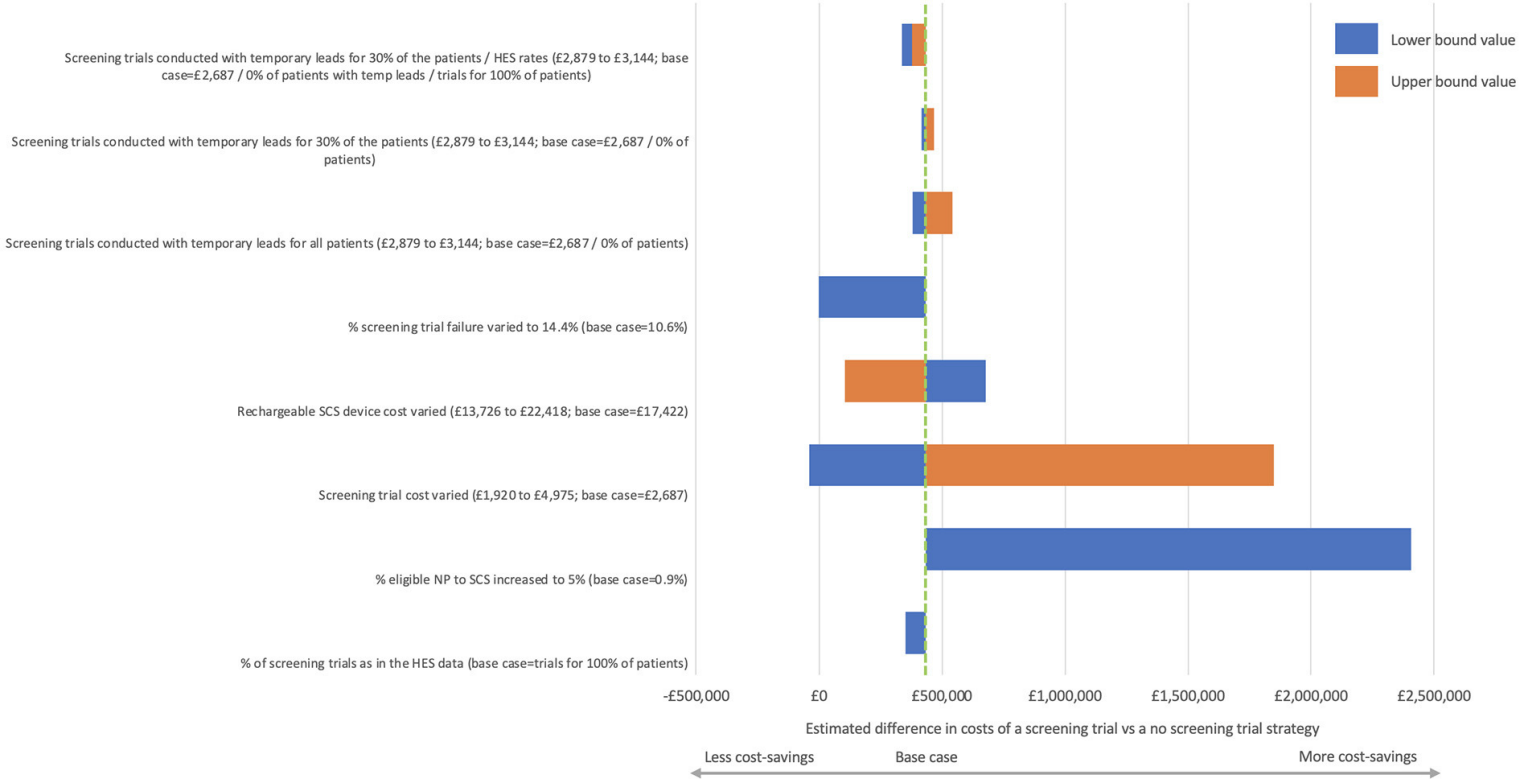
Tornado diagram representing the deterministic sensitivity analyses.

Scenarios considering the use of screening trials using temporary leads that could be removed as an outpatient procedure were also evaluated. A no screening trial strategy remained as cost-saving for scenarios where all the patients have screening trials with temporary leads, 70% of patients have screening trials with permanent leads and 30% of patients with temporary leads (reflecting the rates observed in TRIAL-STIM) (14), and 70% of patients have screening trials with permanent leads and 30% of patients with temporary leads considering the rate of screening trials as observed in the HES data. The tornado diagram illustrates the impact of variations in assumptions in relation to the base case (Figure 1).

## Discussion

The results of this budget impact analysis indicate that discontinuation of screening trials before SCS implantation could result in cost-savings to healthcare providers in England. The base case analysis showed the estimated cost-savings of not conducting a screening trial ranged from £433,189 per year in 2019/20 to near £500,000 per year from 2023/24. The results were robust to sensitivity analysis with screening trials being associated with cost-savings only when the cost of a screening trial was set at its lowest value of £1,920. A screening trial cost of £1,920 is implausible given the list price for the leads alone is often more than this value. The cost-savings observed in sensitivity analysis ranged from £120,042 (rechargeable SCS device cost varied to the upper bound value in 2020/21) to £2,766,139 (proportion of the eligible neuropathic pain population that receive an SCS increased to 5% in 2024/25). A no screening trial strategy results in higher SCS device costs and implant costs as more patients would receive an IPG. However, this is offset by the screening trial costs and unsuccessful screening trial costs incurred in current practice.

The findings from this budget impact analysis support the results of a previous cost analysis that suggested that when using a rechargeable SCS device, a screening trial strategy would become cost-saving when at least 20% of patients had an unsuccessful screening trial (19). In the current study a screening trial prior to SCS implantation would become cost-saving if 14.4% or more of the patients considered for SCS had an unsuccessful screening trial. The difference in the trial conversion rates required is due to updated costs for SCS implantation costs. The exclusion rate of 14.4% is approximately double previous rates observed in routine practice in a single center in England (26).

Within the TRIAL-STIM RCT, data were collected on adverse events and healthcare resource use for patients who had a screening trial followed by SCS implantation and patients that proceeded to SCS implantation without a screening trial. There were no statistically significant differences in these outcomes within the RCT, however the frequency of serious adverse events observed were greater in those patients that had a screening trial prior to SCS implant. Including resource use following the screening trial period would still result in a no screening trial strategy being cost-saving. Estimates for the budget impact implications considering the base case assumptions for the year 2019/20 are presented in Table 5.

**Table 5:** Difference in current practice and a no screening trial strategy for the year 2019/20 including resource use incurred in the first year of SCS.

	**Current practice**	**No Screening trial**
No. patients with SCS	576	576
No. patients with a screening trial	576	-
No. patients with unsuccessful screening trial	61	-
SCS device costs	£8,972,330	£10,035,072
SCS implant costs	£1,996,693	£2,233,194
Screening trial costs	£1,547,520	-
Unsuccessful screening trial costs	£160,282	-
Healthcare resource use	£659,538	£893,260
Adverse events	£12,033	£103,513
Serious adverse events	£276,860	-
Unscheduled visits	£3,597	£36,678
Total	£13,623,688	£13,301,717
Cost difference	£321,970	

SCS, spinal cord stimulation.

The results from this study should be interpreted alongside the findings from the TRIAL-STIM RCT that observed no evidence that a screening trial provides superior patient outcomes or is cost-effective compared to not doing a screening trial (14), with 36-month follow-up results reporting no difference between groups in the long-term (15). In addition, the TRIAL-STIM qualitative study observed that patients were not supportive of SCS screening trials (16). In the context of the COVID-19 pandemic, a no screening trial strategy may fit with ongoing COVID-19 pressures on health services. Overall, the evidence suggests that a screening trial may not represent a good use of healthcare resources and do not necessarily need to be carried out for every patient considered for SCS. However, where there are concerns from medical, psychological or patient perspective on suitability for SCS, a screening trial should be performed with sufficient duration to enable an informed decision on whether to progress to full implantation of the SCS device.

A further potential consequence of screening trials not being compulsory is the release of resources in implanting centers, which may enable an increase in capacity and increase the uptake of SCS in England. The uptake of SCS has been limited with only 0.9% of potential eligible patients with neuropathic pain being considered for SCS (21). The cost-savings would not affect clinicians' income in the NHS but could affect the income of manufacturers of temporary leads. Considering that most, if not all manufacturers of temporary leads also develop permanent leads and SCS devices, the income lost due to not using temporary leads would be offset by an increase in the number of permanent leads and SCS devices. Further, costs for SCS companies where representatives attend the screening trial and implant would be reduced by representatives only having to attend once or in some cases not having to perform aftercare during a home trial.

In the United States, where SCS was first introduced and where SCS trials were first reported (1–3), trials have been required since 1979 by Medicare and, following their example, by third party payers in general. As a result, RCT's such as TRIAL-STIM have not been feasible in the US. Models of US trial cost-effectiveness have relied on clinical data collected in Europe, and eliminating trials has been reported to be a dominant strategy for trials using wireless, externally powered generators, but no models based on US trials have been published (27). Analysis of Truven Marketscan Database up to 2012 has suggested that the trial conversion rate in the US may be as low as 41.4% (28) or 64.7% (29) A difference in practice between England and the US may be that screening trials in England are used as an exclusion test after determining clinical eligibility (i.e., to identify those patients that do not respond to SCS), while screening trials in the US are used as an inclusion test (i.e., screening trial is used as an aid to determine clinical eligibility). Differences between countries may also relate to medical indications of the populations being tested, difference in healthcare setting and payer (e.g., reimbursement not dependent on outcome), physician and patient expectations.

Quality of patient selection will impact on the screening trial failure rates and can be improved by therapeutic education of the patient, a good understanding of the objectives of SCS (including the reduction of drug treatments), psychological evaluation of the patient (to rule out a major depressive disorder, addictive disorders, unreasonable expectations regarding the treatment), a multidisciplinary consultation meeting to decide on the implantation, and a pain physician who makes the final indication for implantation. Under these conditions, it is plausible that 90% or more of the patients who benefit from the test phase would be implanted. A recent retrospective study observed that 86% of patients without compromising clinical or psychosocial factors obtained pain relief ≥50% at 6-months after SCS compared with 60% of patients who were considered to have severe problems identified during multidisciplinary team assessment (30).

### Strengths and weaknesses

We provide a budget impact analysis from the NHS perspective over a 5-year time horizon that reflects a patient population with neuropathic pain in England. We considered several sensitivity analyses to explore the robustness of the results to variations in the parameters of the model. As recommended by a Task Force on good practices for budget impact analysis, we used the simplest design to generate credible and transparent estimates (31).

A limitation of budget impact studies is that health outcomes are not considered in the analysis. The only cost-utility analysis to date evaluating the use of a SCS screening trial compared to a no screening trial strategy concluded that a screening trial was not a cost-effective use of resources (14). This analysis considers the current definition of successful screening trial. It is plausible that redefining screening trials and outcomes used to determine success may improve the ability of a screening trial to observe early response within a shorter time that is predictive of long-term outcome, therefore reducing the costs of screening trials. Reduction in the cost of a screening trial was the only scenario that would enable cost-savings with a screening trial with a 10.6% trial conversion rate. Conversely, improved patient selection (32, 33) resulting in 100% conversion rates from trial to implant, would render this discussion resolved, where no screening trials would be required.

## Conclusions

The findings from this budget impact analysis suggest that not conducting compulsory screening trials of SCS would result in considerable cost-savings to the healthcare system in England. A screening trial would only result in cost-savings at the lower cost for a screening trial or when at least 14.4% of patients considered for SCS have an unsuccessful trial, approximately double the rate observed recently in routine practice in England.

## Data availability statement

The original contributions presented in the study are included in the article/supplementary material, further inquiries can be directed to the corresponding author/s.

## Ethics statement

Ethical review and approval was not required for the study on human participants in accordance with the local legislation and institutional requirements. Written informed consent for participation was not required for this study in accordance with the national legislation and the institutional requirements.

## Author contributions

RD, SN, RH, MB, JB, JE, RT, and SE were responsible for the original proposal and securing funding for the project. RD and SN acquired the aggregate Hospital Episode Statistics data. RH conducted the analysis of the data with assistance from RD. RD and RH interpreted the data and wrote the first draft of the manuscript. All authors contributed to and approved the final version of the manuscript.

## Funding

This research was funded by the National Institute for Health Research (NIHR) Policy Research Programme (PRP) (project number: NIHR201444). The funding source had no role in the study design, data collection, data analysis, interpretation of data, writing of the manuscript, approval or decision to submit the manuscript for publication.

## Conflict of interest

RD was an employee of Saluda Medical. He has previously received consultancy fees from Boston Scientific Corp, Mainstay Medical, Medtronic Ltd and Saluda Medical. AG has received honoraria for consulting as well as advisory board meetings for Nevro Corp, Boston Scientific Corp and Abbott. ST has received consultancy fees from Boston Scientific Corp, Mainstay Medical and Saluda Medical. He has received department research funding from the National Institute of Health Research, Boston Scientific Corp, Saluda Medical and Mainstay Medical. GB has a consulting agreement and is on the advisory board for Nevro Corp, Nalu Medical Inc., Abbott and Boston Scientific Corp. RN serves as an unpaid officer of the non-profit Neuromodulation Foundation Inc, to which (like his former employers Johns Hopkins University and Sinai Hospital) grants and support have been provided by Abbott, Boston Scientific Corp, Medtronic, Inc., Nevro Corp, Nuvectra, and Stimwave, Inc. He receives royalties from Abbott and consulting fees and royalties from Nuvectra. His wife holds shares in Stimwave, Inc. RT has received consultancy fees from Medtronic Ltd, Nevro Corp and Saluda Medical. SE has received consultancy fees from Medtronic Ltd, Mainstay Medical, Boston Scientific Corp, and Abbott. He has received department research funding from the National Institute of Health Research, Medtronic Ltd. and Nevro Corp. The remaining authors declare that the research was conducted in the absence of any commercial or financial relationships that could be construed as a potential conflict of interest.

## Publisher's note

All claims expressed in this article are solely those of the authors and do not necessarily represent those of their affiliated organizations, or those of the publisher, the editors and the reviewers. Any product that may be evaluated in this article, or claim that may be made by its manufacturer, is not guaranteed or endorsed by the publisher.

## Author disclaimer

The views expressed in this publication are those of the authors and do not necessarily reflect those of the PRP programme, NIHR, NHS, or the Department of Health and Social Care.
